# Long non-coding RNA ncRuPAR regulates gastric cancer cell proliferation and apoptosis via phosphoinositide 3-kinase/protein kinase B signaling

**DOI:** 10.7150/ijms.76664

**Published:** 2022-10-17

**Authors:** Renjie Xu, Jiahui Yu, Shangjin Song, Dazhi Sun, Lijuan Xiu, Jinyu Xu, Jing Zhao, Xuan Liu, Qing Ji, Xiaoqiang Yue

**Affiliations:** 1 Department of Traditional Chinese Medicine, Changzheng Hospital, Naval Medical University, Shanghai 200003, China.; 2 School of Traditional Chinese Medicine, Naval Medical University, Shanghai, 200433, China.; 3 Strategic Support Force Xingcheng Special Duty Sanatorium, Xingcheng 125100, Liaoning Province, China.; 4 Cancer Institute, Shuguang Hospital, Shanghai University of Traditional Chinese Medicine, Shanghai 201203, China.

**Keywords:** ncRuPAR, gastric cancer, cell proliferation, apoptosis, PI3K/Akt signaling

## Abstract

**Objective:** To determine the effect and mechanism of the long non-coding RNA (lncRNA) ncRuPAR (non-protein coding RNA, upstream of coagulation factor II thrombin receptor [F2R]/protease-activated receptor-1 [PAR-1]) in human gastric cancer.

**Methods:** HGC-27-ncRuPAR overexpression and MGC-803-ncRuPAR-RNAi knockdown gastric cancer cell lines were established. We assessed the effect of ncRuPAR on cell proliferation, apoptosis, migration, and invasion using Cell Counting Kit 8, flow cytometry, scratch and transwell assays, respectively. Differentially expressed genes in HGC-27-ncRuPAR overexpression and HGC-27-empty vector cell lines were identified using Affymetrix GeneChip microarray analysis. Ingenuity Pathway Analysis (IPA) of the microarray results was subsequently conducted to identify ncRuPAR-enriched pathways, followed by validation using real time-quantitative PCR (RT-qPCR). As one of the top enriched pathways, phosphoinositide 3-kinase (PI3K)/protein kinase B (Akt) signaling pathway was further examined by western blotting to determine its role in ncRuPAR-mediated regulation of gastric cancer pathogenesis.

**Results:** ncRuPAR inhibited human gastric cancer cell proliferation and induced G1/S phase arrest and apoptosis, but did not affect migration or invasion *in vitro*. Overexpression of ncRuPAR *in vitro* was found to inhibit its known target PAR-1, as well as PI3K/Akt signaling. The downstream targets of PI3K/Akt, cyclin D1 was downregulated, but there was no change in expression level of B-cell lymphoma 2 (Bcl-2).

**Conclusions:** We showed that lncRNA-ncRuPAR could inhibit tumor cell proliferation and promote apoptosis of human gastric cancer cells, potentially by inhibiting PAR-1, PI3K/Akt signaling, and cyclin D1. The results suggest a potential role for lncRNAs as key regulatory hubs in GC progression.

## Introduction

Gastric cancer is prevalent worldwide and has a higher incidence in males than females 1. Although there is no lack of treatment options including a combination of surgery, chemotherapy, radiotherapy, immunotherapy and/or adjuvant therapy 2, treatment efficacy and patient survival rates depend on tumor staging, occurrence of metastasis, drug resistance and tumor recurrence 1-3. Disease prognosis has vastly improved with a better understanding of disease etiology and advancements in early diagnostics and treatment methods 1,4. The risk of gastric cancer can be reduced by effective prevention measures against *Helicobacter pylori* infection, and adopting a healthy diet and lifestyle 1. However, due to inherent tumor heterogeneity and late diagnoses, gastric cancer remains one of the top causes of cancer-related deaths globally 5,6. Deeper insight into the mechanisms underlying its pathogenesis could facilitate early diagnosis and development of improved interventions.

Long non-coding RNAs (lncRNA) of more than 200 nucleotides in length have well-reported roles in regulating the tumorigenesis and progression of many cancers, including gastric cancer 7,8. In addition, they can modulate drug resistance and have been proposed as potential prognostic markers and therapeutic targets 7-11. The lncRNA ncRuPAR (non-protein coding RNA, upstream of coagulation factor II thrombin receptor [F2R]/protease-activated receptor-1 [PAR-1]) located on chromosome 5 has been shown to regulate gastric cancer malignancy, wherein its downregulation can promote angiogenesis, invasion, metastasis and progression 7,12. The high levels of the G-protein coupled receptor PAR-1, but low levels of ncRuPAR, observed in gastric cancer suggests the inhibition of PAR-1 by ncRuPAR^12^. Suppression of ncRuPAR expression has also been shown to contribute to hepatocellular carcinoma pathogenesis and progression. Given that the molecular mechanism of ncRuPAR is poorly characterized, this study was conducted to elucidate its regulatory effects in gastric cancer.

After preliminary screening of ncRuPAR expression levels in four human gastric cancer cell lines (HGC-27, MKN-45, MGC-803 and SGC-7901) and human gastric mucosal cells (GES-1) by quantitative PCR (qPCR), MGC-803 and HGC-27 human gastric cancer cells were chosen to establish stable lentiviral ncRuPAR knockdown and overexpression cell lines, respectively. These cell lines were subsequently used for *in vitro* experiments to investigate the effect of ncRuPAR expression on tumor cell proliferation, cell cycle, apoptosis, migration, and invasion in human gastric cancer cells. Methods included flow cytometry, Cell Counting Kit-8 (CCK-8), scratch, and transwell assays for the *in vitro* experiments. Affymetrix GeneChip microarray and ingenuity pathway analysis (IPA) followed by qPCR and western blot analysis were subsequently performed to examine the molecular mechanism of ncRuPAR in human gastric cancer. Given that PAR-1 can activate phosphoinositide 3-kinase (PI3K)/protein kinase B (Akt) signaling 13-18, it was speculated that the PI3K/Akt pathway could be a possible downstream target of ncRuPAR and this study explored their relationship in gastric cancer. These findings provide preliminary insight into the potential underlying mechanism of ncRuPAR-mediated regulation of human gastric cancer cell proliferation and apoptosis.

## Materials and Methods

### Cell lines and culture

Human gastric cancer cell lines (HGC-27, MKN-45, MGC-803, SGC-7901) and human gastric mucosal cells (GES-1) were purchased from the Cell Resource Center of Shanghai Institutes for Biological Sciences, China. Cell lines were maintained in RPMI-1640 medium containing 10% fetal bovine serum (FBS), penicillin (100 U/mL), and streptomycin (100 mg/mL) (Invitrogen, USA) at 37 ºC with 5% CO_2_.

### Establishment of stable ncRuPAR RNAi knockdown and overexpression human gastric cancer cell lines

The expression level of ncRuPAR was detected in four human gastric cancer cell lines (HGC-27, MKN-45, MGC-803, SGC-7901) and human gastric mucosal cells (GES-1) by qPCR. As ncRuPAR was lowly expressed in HGC-27 cells and highly expressed in MGC-803 cells, these two cell lines were used to establish stable overexpression (ncRuPAR) and RNAi knockdown (ncRuPAR-RNAi) cell lines, respectively. MGC-803 and HGC-27 cell lines were infected with lentiviral ncRuPAR RNAi knockdown (LV-ncRuPAR-RNAi [54387-1]), overexpression LV-HGC-27-ncRuPAR (15034-1), empty LV-ncRuPAR-RNAi-CON, and LV-ncRuPAR-CON plasmids. Lentiviral infection reagents were purchased from Shanghai Genechem Co. Ltd. Puromycin (2 μg/mL) selection after 72 h allowed us to confirm the establishment of stable MGC-803 ncRuPAR RNAi knockdown and HGC-27 ncRuPAR overexpression cell lines by qPCR. Four lentiviral cell lines were obtained: MGC803-ncRuPAR-RNAi and MGC803-empty vector, as well as HGC27-ncRuPAR and HGC27-empty vector. These four lentiviral cell lines together with uninfected MGC-803 and HGC-27 cells (control) were used for subsequent experiments.

### Real-time quantitative PCR

The relative expression levels of ncRuPAR in HGC-27, MKN-45, MGC-803, SGC-7901 (four gastric cancer cell lines) were determined by real-time qPCR. Differentially expressed genes targeted by ncRuPAR were also confirmed by real-time qPCR. Total RNA was isolated using TRIzol reagent (Invitrogen, USA) according to the manufacturer's instructions. The quality and quantity of RNA samples were assessed using a microplate spectrophotometer (Multiskan MK3, Thermo Fisher Scientific, USA). Reverse transcription into complementary (c)DNA was performed using the PrimeScript RT Master Mix kit (Takara, Dalian, China). The relative gene expression levels were determined by real-time qPCR using the SYBR Premix Ex TaqTM (Takara, Japan) and specific primers in the IQ5 Multicolor Real-Time PCR Detection system (Bio-Rad Laboratories, China). The primers were listed in Table 1. And the relative ncRuPAR expression levels were determined using the 2^-ΔΔCT^ method 19.

### CCK-8 assay

The effect of ncRuPAR on human gastric cancer cell proliferation was determined using the CCK-8 assay (Sigma, USA). Briefly, cells were seeded into 96-well plates at 1x10^4^ cells/well. When cell concentration reached 60%, the medium was replaced with fresh RPMI-1640 medium containing 10% FBS, penicillin (100 U/mL), and streptomycin (100 mg/mL), and cells were incubated for 48 h. Absorbance was read at 450 nm using a microplate enzyme-linked immunosorbent assay reader (Wellscan MK 3, Labsystem Dragon, USA). The experiment was performed in triplicate, with five wells per experiment. The IC_50_ (half maximal inhibitory concentration) values was estimated using the following formula: IC_50_=lg-1[Xm-i(ΣP-0.5)] 20.

### Colony formation assay

A cell monolayer in the logarithmic growth phase was digested with 0.25% trypsin and sorted into individual cells in RPMI 1640 medium containing 10% FBS. The cell suspension (200 cells/well) was then inoculated into 12-well plates and gently rotated to ensure uniform spreading of the cells. The cells were cultured at 37 ºC, with 5% CO_2_ and 100% saturated air for two weeks. Upon the appearance of colonies, the culture was halted. The supernatant was discarded, and the cells were washed twice with phosphate buffered saline (PBS). The colonies were fixed in 5 mL pure methanol or 1:3 acetic acid for 15 min before staining with Giemsa solution for 10 min. Giemsa solution was then slowly rinsed off with running water and colonies were left to air-dry. Colonies were counted under a microscope (Olympus, Japan) at a low magnification (4x). Only colonies larger than 10 cells were counted. Triplicates were performed.

### Flow cytometry

Flow cytometry was performed to determine the effect of ncRuPAR on apoptosis and the cell cycle of human gastric cancer HGC-27 and MGC-803 cell lines. Cells (1×10^5^ cells/well) were seeded in six-well plates with annexin-binding buffer and were double-stained using 5 µL annexin V-FITC and 1 µL propidium iodide (PI) (BD Biosciences, USA). Next, flow cytometric analysis was performed (FACS Calibur, BD Biosciences) to detect apoptotic cells. For cell cycle analysis, cells were fixed with 70% ethanol overnight and stained with PI (0.1 mg/mL) in the presence of ribonuclease A (Takara) for 30 min at room temperature. The cell cycle distribution was examined by flow cytometry, and results were analyzed using FlowJo software (Tree Star Corp., USA).

### Scratch assay

Scratch assay was performed to assess cell migration. HGC-27 and MGC-803 cell lines were inoculated into a six-well plate (1×10^5^ cells/well) and cultured at 37 ºC under 5% CO_2_ overnight. A straight line was draw on the bottom of the six-well plate using a marker and a ruler. When cell concentration reached 100%, a 10 µL pipette tip was used to scratch horizontal lines into the cell monolayers (perpendicular to the line drawn on the bottom). Cells were carefully rinsed with PBS for three times to remove dislodged cells, and then serum-free medium was added to the well. Cell culture was continued at 37 ºC under 5% CO_2_ and the cells were continuously monitored. Images were captured after 72 h using an inverted microscope (Olympus, Japan).

### Transwell invasion assay

Matrigel transwell assay was performed to assess the effect of ncRuPAR on human gastric cancer cell invasion. Serum-free culture media was added to the upper chambers and culture media containing 600 μg fibronectin and 10% FBS was added to the lower chambers of a 24-well Transwell plate (Corning, USA). HGC-27 and MGC-803 cell suspensions (4×10^5^ cells/mL; 150 μg/well) in RPMI-1640 medium were inoculated in the upper chambers after removal of the serum-free culture media. After 24 hours, the chambers were removed. The non-invasive cells in the upper chamber were gently removed with a cotton swab, and the chamber was rinsed several times with PBS. The membrane was fixed with 95% ethanol for 10 min and then stained with crystal violet for 20 min to stain the cells that had invaded. Images of each chamber, with three random visual fields of view, were captured using an inverted microscope. Triplicates were performed.

### Microarray and IPA analysis

Total RNA was extracted from HGC-27-ncRuPAR and HGC-27-empty vector human gastric cancer cell lines with TRIzol reagent for microarray analysis using an Affymetrix GeneChip and Gene Array Scanner (Thermo Fisher Scientific, USA). Supplementary material 1 shows a flowchart for microarray data processing and analysis. Data analysis was performed using IPA (Ingenuity Pathway Analysis) software. Genes with fold change absolute values >1.5 and FDR < 0.05 were deemed to be differentially expressed. Hierarchical clustering of differentially expressed genes was presented in a heatmap. These differentially expressed genes were then further analyzed by IPA analysis to identify the possible target genes and signaling pathways of ncRuPAR that may regulate human gastric cancer cell proliferation and apoptosis. The microarray results were uploaded into Qiagen's IPA system and were compared with the ingenuity pathway knowledge base. The differentially expressed genes were categorized according to their respective canonical pathways, diseases and functions, and gene interaction networks. The signaling pathways underwent retrograde sorting by -log(P-value), with more significantly enriched pathways having lower P values. The z scores, which indicate the degree of pathway activation or inhibition, were calculated. Scores of z > 2 indicate pathway activation while z < -2 indicate pathway inhibition. The ratio of the number of differentially expressed genes with respect to the total number of genes in a particular signaling pathway was also calculated.

### Western blot analysis

Total protein was extracted from HGC-27-ncRuPAR, HGC-27-empty vector, MGC-803-ncRuPAR-RNAi, and MGC-803-empty vector stable lentiviral cell lines using RIPA lysis buffer and protein concentration was determined using a BCA assay. Proteins were separated by sodium dodecyl sulphate-polyacrylamide gel electrophoresis (SDS-PAGE) and separated bands were transferred onto a polyvinylidene fluoride (PVDF) membrane. The membrane was incubated with primary antibodies against β-actin(4970T), PAR-1(79109S), PI3K(13666S), phosphorylated (p)-Akt(4060T), cyclin D1(55506T) or Bcl-2(15071T) (1:1,000 dilution; Cell Signaling Technology Inc., USA) overnight at 4 ºC. Subsequently, membranes were incubated with a goat anti-rabbit (A0208) or anti-mouse (A0192) horseradish peroxidase (HRP)-conjugated secondary antibody (1:2,000 dilution; Beyotime Biotechnology Inc., ShangHai, China) at room temperature for 1.5 h. Bands were visualized using an enhanced chemiluminescence reagent. Densitometric analysis was performed using Scion Imaging software (ScionCorp., USA). β-actin (0.1 µg/mL) was used as the internal loading control.

### Statistical analysis

Statistical analysis was performed using SPSS software version 20 (IBM Corporation, Armonk, NY, USA) and GraphPad Prism 5 (GraphPad Software, La Jolla, CA, USA). Data of at least three independent experiments were expressed as the mean ± standard deviation (SD). Significance was determined using a two-tailed paired Student's t test, or one-way analysis of variance (ANOVA) followed by S-N-K (S) or Dunnett's test as appropriate. p<0.05 was considered to be statistically significant.

## Results

### Expression of ncRuPAR in ncRuPAR RNAi knockdown and overexpression gastric cancer cell lines

**Figure 1:**
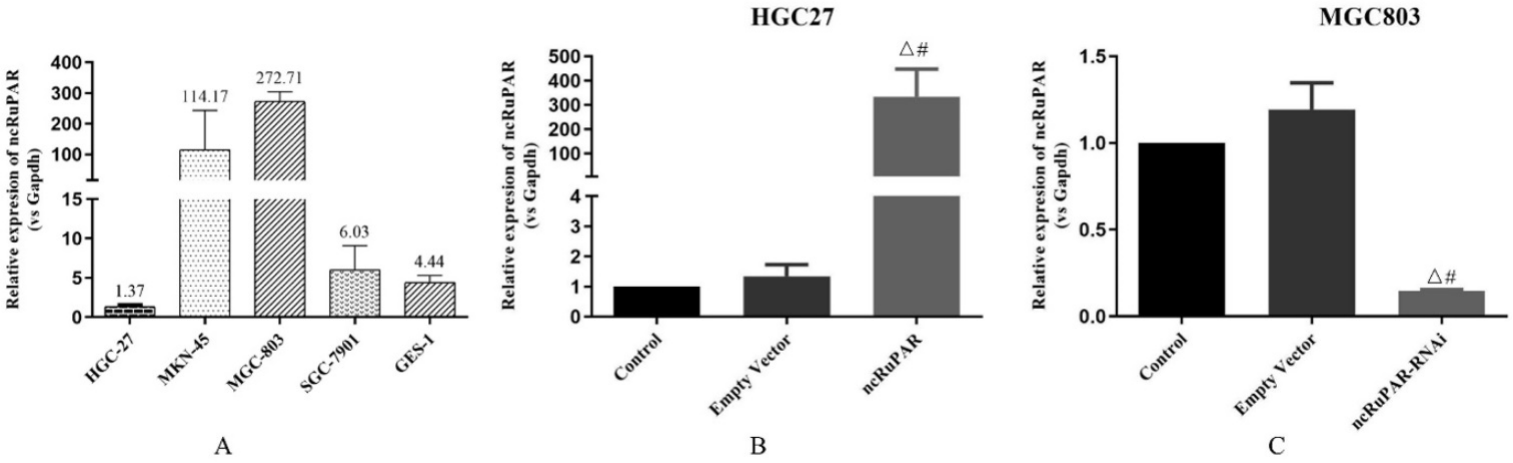
** Establishment of stable ncRuPAR RNAi knockdown and overexpression in human gastric cancer cell lines. (A)** qPCR results of ncRuPAR expression in different gastric cancer cell lines. Establishment of stable **(B)** HGC-27-ncRuPAR overexpression and **(C)** MGC-803-ncRuPAR RNAi knockdown cell lines. ^#^: *p* < 0.05 vs control; Δ: *p* < 0.05 vs empty vector. HGC-27 and MGC-803 cell lines not infected with lentiviral constructs (control); infected with lentiviral empty vector constructs (empty vector); ncRuPAR overexpression (ncRuPAR); ncRuPAR RNAi knockdown (ncRuPAR-RNAi).

The expression levels of ncRuPAR were detected in HGC-27, MKN-45, MGC-803, SGC-7901, (four gastric cancer: 1.37 ± 0.20; 114.17 ± 129.61; 272.71 ± 31.04; 6.03 ± 3.05; respectively) and GES-1 (normal gastric epithelium; 2.44 ± 0.85) cell lines using qPCR (Fig. 1A). Given the low expression of ncRuPAR in HGC-27 cells and high expression in MGC-803 cells, the former was selected to establish the stable ncRuPAR overexpression cell line and the latter to establish the stable ncRuPAR RNAi knockdown cell line. qPCR confirmed the establishment of stable cell lines (Fig. 1B-C; *p* < 0.05).

### ncRuPAR inhibits human gastric cancer cell proliferation

**Figure 2:**
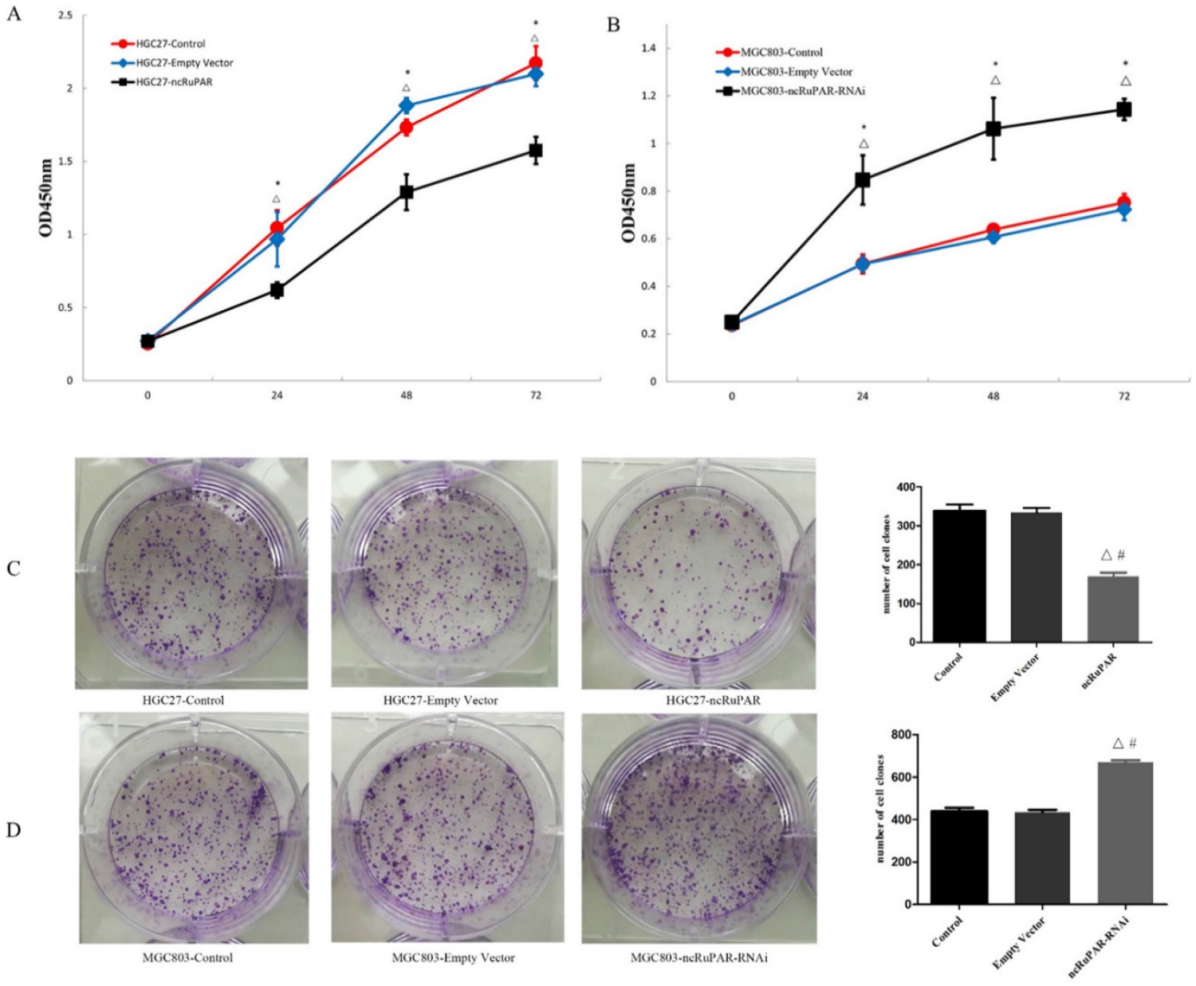
** ncRuPAR inhibits human gastric cancer cell proliferation.** CCK-8 assay profiles of **(A)** HGC-27 (control, empty vector, ncRuPAR) and **(B)** MGC-803 (control, empty vector, ncRuPAR-RNAi) cell lines. **(C-D)** Colony formation assay images and **(E-F)** corresponding histograms of **(C, E)** HGC-27 and **(D, F)** MGC-803cell lines. ^#^: *p* < 0.05 vs control; △: *p* < 0.05 vs empty vector.

The effect of ncRuPAR knockdown or overexpression on human gastric cancer cell proliferation was assessed using CCK-8 and colony formation assays. It was found that ncRuPAR overexpression decreased HGC-27 cell proliferation at 24, 48 and 72 h time points (Fig. 2A; *p* < 0.05), while ncRuPAR knockdown increased MGC-803 cell proliferation (Fig. 2B; *p* < 0.05). The effect of ncRuPAR on human gastric cancer cell proliferation was supported by the results of the colony formation assay. Fewer colonies formed in ncRuPAR knockdown cells, whereas more colonies formed when ncRuPAR was overexpressed (Fig. 2C-D; *p* < 0.05).

### ncRuPAR induces G1/S phase arrest and increases human gastric cancer cell apoptosis

**Figure 3:**
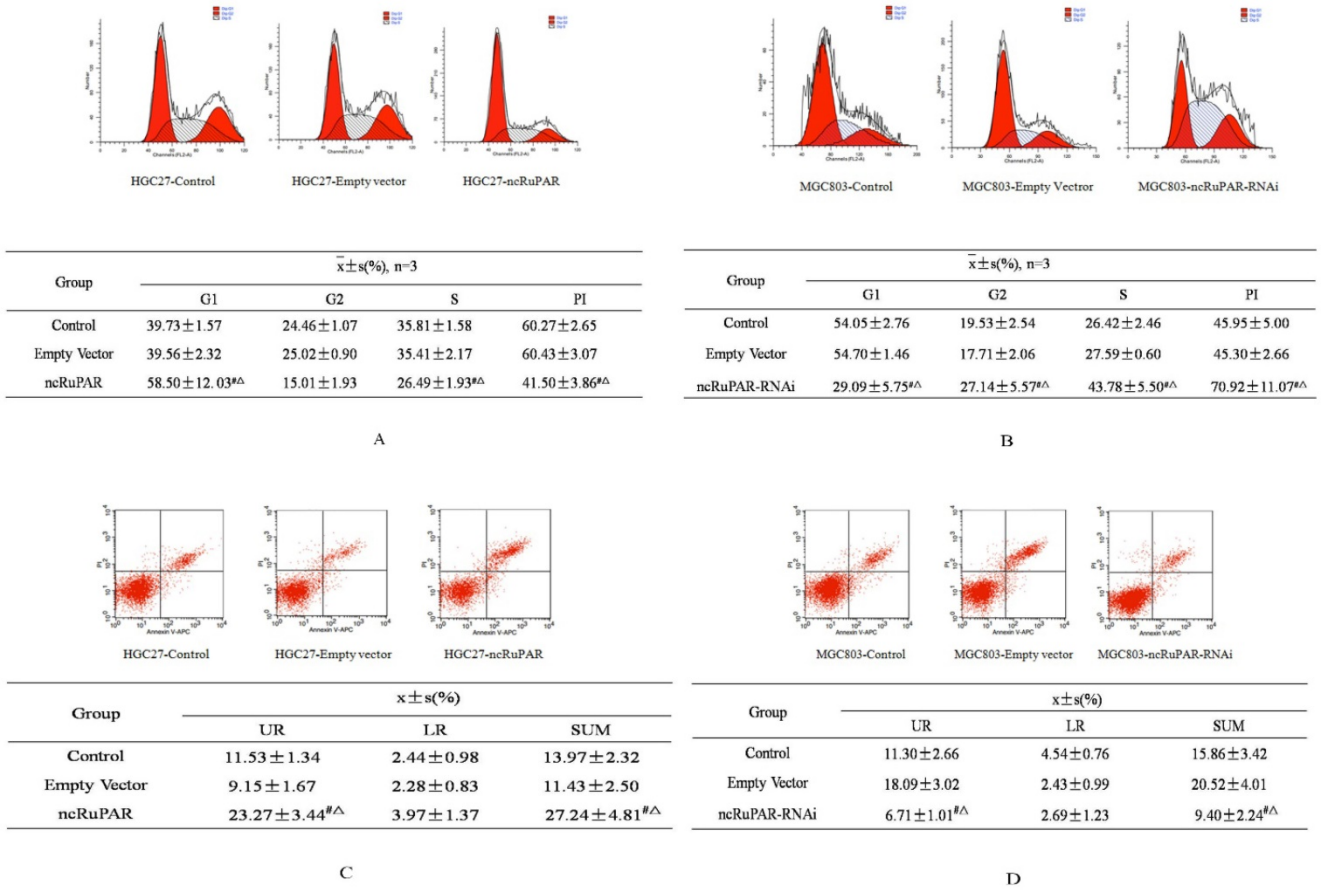
** ncRuPAR inhibits G1/S phase transition and increases apoptosis of human gastric cancer cells.** Flow cytometric profiles of the effect of ncRuPAR on **(A-B)** cell cycle and **(C-D)** apoptosis in **(A, C)** HGC-27 (control, empty vector and ncRuPAR) and **(B, D)** MGC-803 (control, empty vector and ncRuPAR-RNAi) cell lines. ^#^: *p* < 0.05 vs control; △: *p* < 0.05 vs empty vector.

Next, the effect of ncRuPAR on cell cycle and apoptosis of human gastric cancer cells was determined by flow cytometry. The results revealed that ncRuPAR inhibited the G1/S phase transition as well as decreased cell division and the proliferation index (PI) in HGC-27 cells overexpressing ncRuPAR (Fig. 3A; *p* < 0.05). Conversely, there were fewer cells in G1 phase and more cells in G2 and S phases in ncRuPAR RNAi knockdown MGC-803 cells (Fig. 3B; *p* < 0.05). Annexin V/PI staining revealed increased apoptosis in HGC-27 cells that overexpressed ncRuPAR (Fig. 3C; *p* < 0.05; ncRuPAR: 26.23 ± 0.78 vs control: 13.52 ± 0.86 and empty vector: 11.43 ± 0.49) and decreased apoptosis of RNAi knockdown ncRuPAR MGC-803 cells (Fig. 3D; *p* < 0.05; ncRuPAR-RNAi: 9.37 ± 0.86 vs control: 16.69 ± 0.75 and empty vector: 17.98 ± 1.82).

### ncRuPAR does not affect human gastric cancer cell migration and invasion

**Figure 4:**
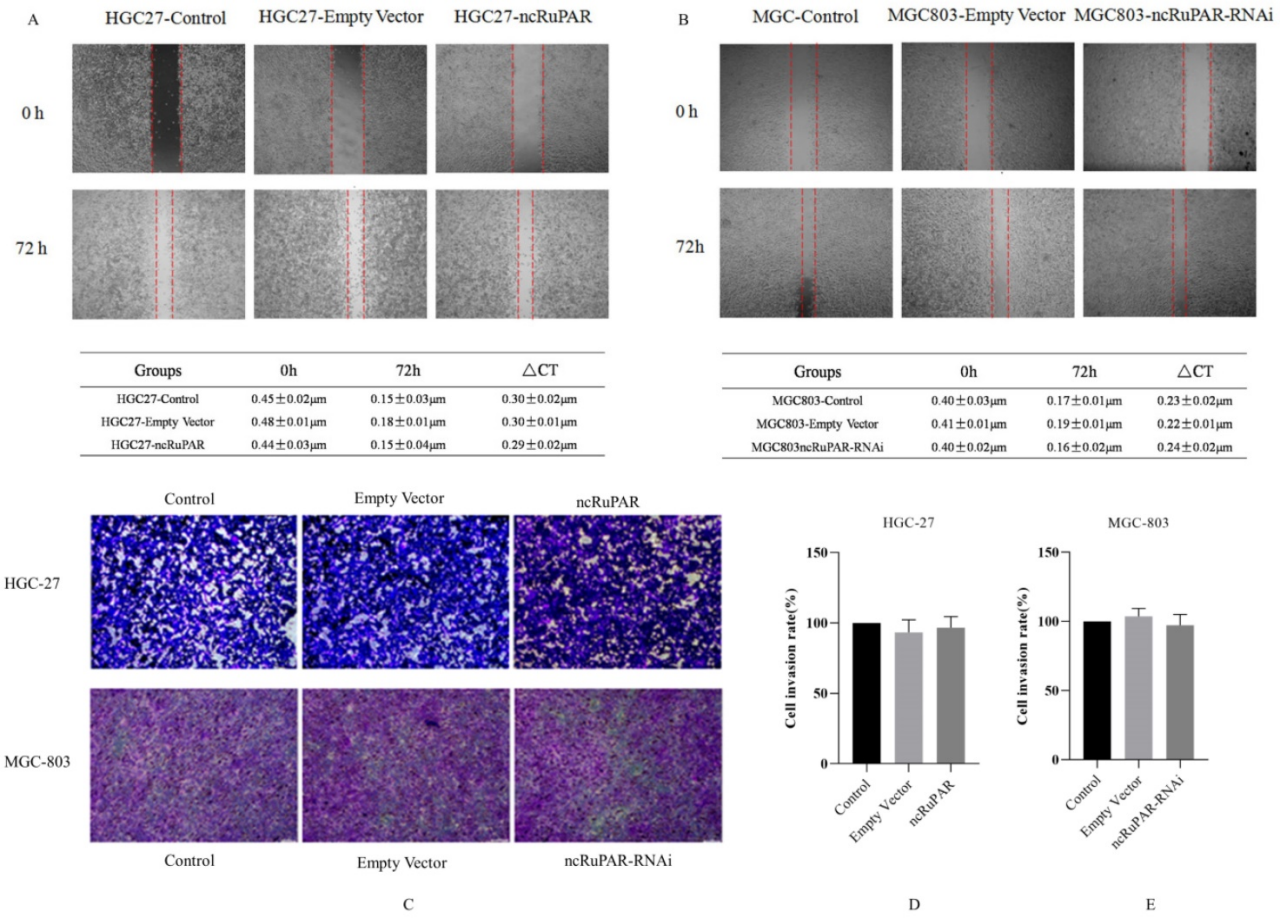
** ncRuPAR does not affect the migration and invasion of human gastric cancer cells (n = 3).** Scratch assay images of **(A)** HGC-27 (control, empty vector and ncRuPAR) and **(B)** MGC-803 (control, empty vector and ncRuPAR-RNAi) cell lines at the 72 h time point showed that ncRuPAR expression had no effect on the migration of these cells. Transwell assay **(C)** images and **(D-E)** corresponding histograms of the invasion rate of (C [top row], D) HGC-27 (control, empty vector and ncRuPAR) and (C [bottom row], E) MGC-803 (control, empty vector and ncRuPAR-RNAi) cell lines.

Scratch and transwell assays were performed to determine the effect of ncRuPAR on migration and invasion of human gastric cancer cells. The scratch assay revealed that there was no change in the migration ability of HGC-27 and MGC-803 cells upon ncRuPAR overexpression or knockdown (Fig. 4A-B; *p* < 0.05). Similarly, the transwell assay revealed that the ncRuPAR expression level (either overexpression or RNAi knockdown) did not affect the invasion ability of HGC-27 (ncRuPAR: 96.67 ± 7.85% and empty vector: 93.33 ± 9.03% vs control) and MGC-803 (ncRuPAR-RNAi: 97.34 ± 7.76% and empty vector: 103.67 ± 5.79% vs control) gastric cancer cells (Fig. 4C-E; *p* < 0.05).

### Microarray profiling of differentially expressed genes and associated signaling pathways targeted by ncRuPAR

**Figure 5:**
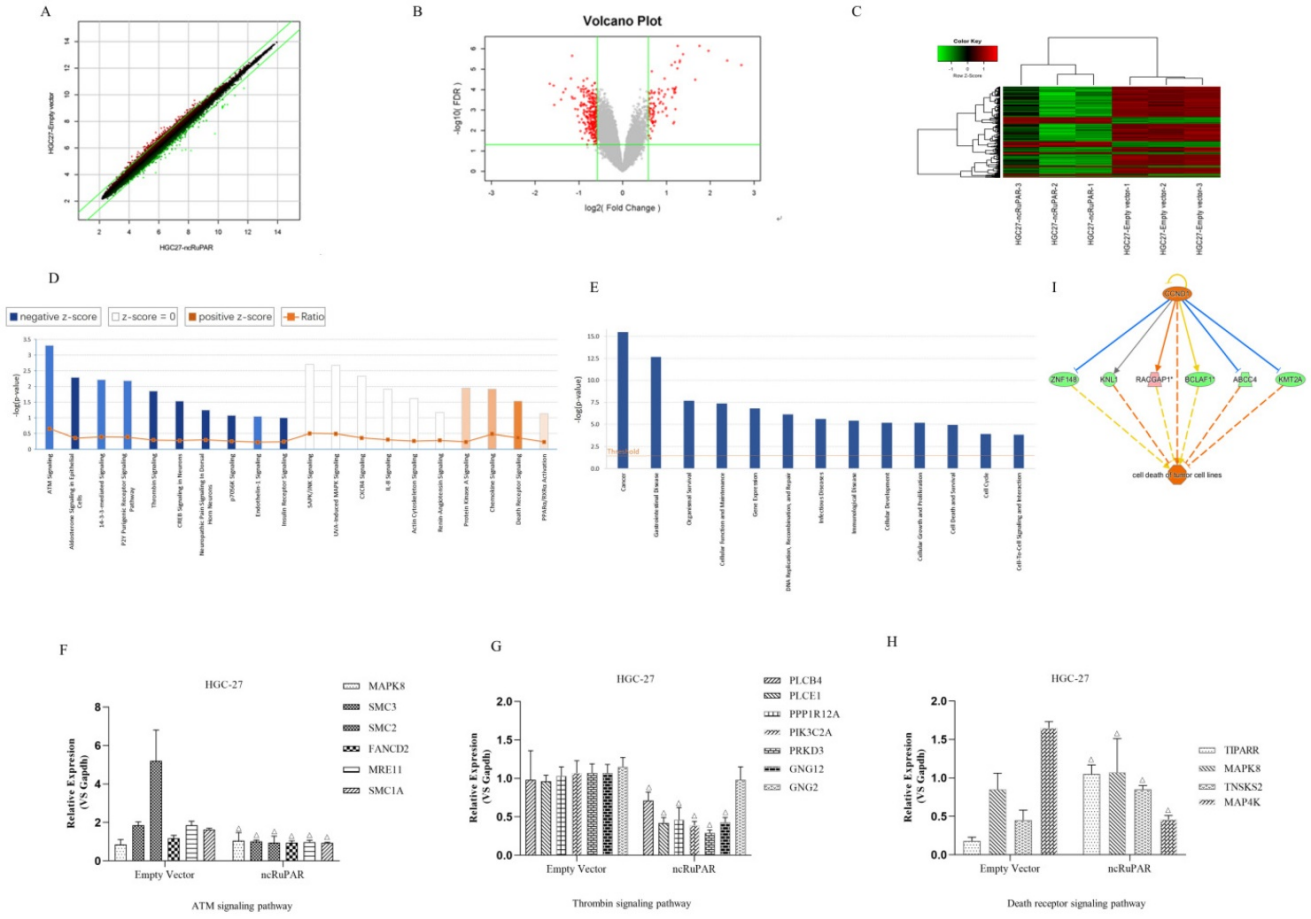
** Microarray and IPA analysis of ncRuPAR target genes and signaling pathways in HGC-27 human gastric cancer cells.** Affymetrix GeneChip microarray **(A)** scatter plot **(B)** volcano plot and **(C)** heatmap of differentially expressed genes in HGC-27-ncRuPAR cells compared with HGC-27-empty vector cells. Upregulated and downregulated genes are shown in green and red, respectively. IPA -log(P-value) analysis profiles of **(D)** enriched disease and function and **(E)** enriched signaling pathways in HGC-27 cells that overexpress ncRuPAR. **(F-H)** qPCR validation of differentially expressed genes from **(F)** ATM signaling, **(G)** thrombin signaling and **(H)** death receptor signaling pathways. **(I)** Gene regulatory network map of the death receptor signaling pathway from IPA analysis showing possible downstream targets of cyclin D1 (CCND1 gene) that could promote tumor apoptosis.

A total of 38,079 effective spectra out of 49,395 mass spectra were collected. There were 295 differentially expressed genes identified from Affymetrix GeneChip microarray profiling of HGC-27-ncRuPAR and HGC-27-empty vector cell lines (Fig. 5A-C). Of these, there were 61 upregulated genes and 234 downregulated genes. IPA analysis of the microarray results were subsequently performed and revealed significant functional enrichment in cell proliferation and apoptosis of cancer cell lines (z > 2 indicating upregulation), and energy homeostasis and cell proliferation of tissues and fibroblasts (z < -2 indicating downregulation) (Fig. 5D). Moreover, it was noted that seven of the top 30 signaling pathways had known associations with tumorigenesis (Fig. 5E; ATM[Ataxia Telangiectasia Mutated] Signaling, P2Y Purigenic Receptor Signaling Pathway, 14-3-3-mediated signaling, Protein Kinase A Signaling, Signaling by Rho Family GTPases, Thrombin Signaling and Death Receptor Signaling). Of the seven cancer-related pathways identified from IPA analysis, 16 ncRuPAR-targeted genes were validated by qPCR and were involved in ATM signaling (six genes; upregulated: MAPK8 (Mitogen-Activated Protein Kinase 8); downregulated: SMC3 (Structural Maintenance Of Chromosomes 3), SMC2 (Structural Maintenance Of Chromosomes 2), FANCD2(FA Complementation Group D2), MRE11(MRE11 Homolog, Double Strand Break Repair Nuclease) and SMC1A(Structural Maintenance Of Chromosomes 1A)), thrombin signaling (seven genes; upregulated: GNG2 (G Protein Subunit Gamma 2); downregulated: PLCB4 (Phospholipase C Beta 4), PLCE1 (Phospholipase C Epsilon 1), PPP1R12A (Protein Phosphatase 1 Regulatory Subunit 12A), PIK3C2A (Phosphatidylinositol-4-Phosphate 3-Kinase Catalytic Subunit Type 2 Alpha), PRKD3 (Protein Kinase D3) and GNG12(G Protein Subunit Gamma 12)) and death receptor signaling (four genes; upregulated: TIPARP (TCDD Inducible Poly (ADP-Ribose) Polymerase), MAPK8, TNKS2 (Tankyrase 2), and MAP4K4 (Mitogen-activated protein kinase kinase kinase kinase-4) pathways. The former two pathways were predicted to be downregulated (z > 2) while the latter was upregulated (z < -2) in HGC-27 ncRuPAR overexpression cells (Fig. 5E). The microarray results for all genes except GNG2 and MAP4K4 (Fig. 5F-H) were confirmed by qPCR. IPA analysis of the death receptor signaling pathway suggests that cyclin D1 (encoded by the CCND1 gene) may play an upstream role in gastric cancer cell apoptosis via ABCC4 (ATP binding cassette subfamily C member 4), BCLAF1 (BCL2 Associated Transcription Factor 1), KMT2A (Lysine Methyltransferase 2A), KNL1 (Kinetochore Scaffold 1), RACGAP1 (Rac GTPase activating protein 1), and ZNF148 (Zinc Finger Protein 148) (Fig. 5I). The fold-change values from both microarray and qPCR validation were listed in Table 2.

### ncRuPAR inhibits PI3K/Akt signaling *in vitro*

**Figure 6:**
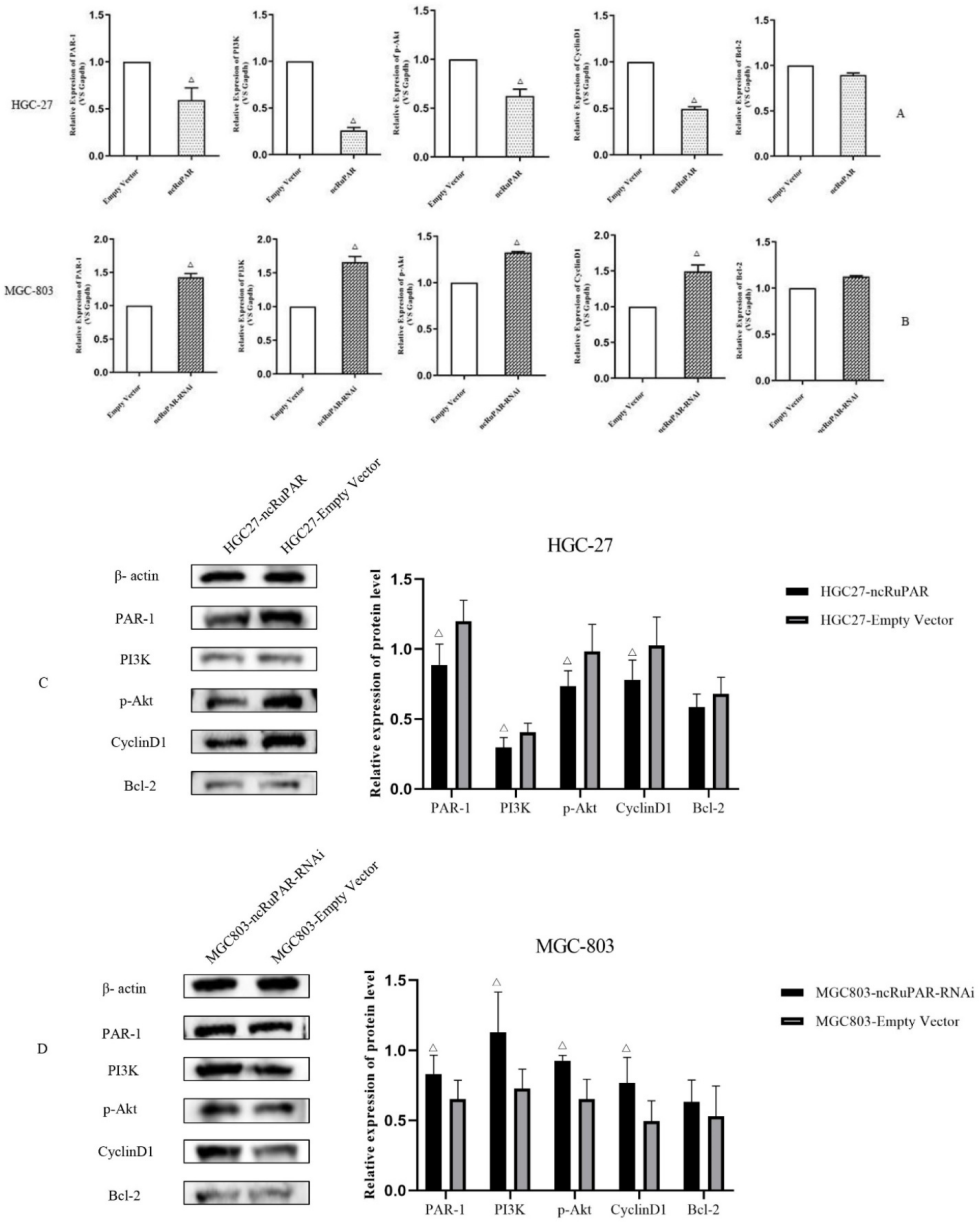
** ncRuPAR inhibits PI3K/Akt signaling pathway activation in human gastric cancer cells *in vitro*. (A-B)** qPCR mRNA profiles of par-1 and PI3K/Akt signaling pathway factors, PI3K, AKT1, CCND1 and BCL-2, in (A) HGC-27 (ncRuPAR and empty vector) and (B) MGC-803 (ncRuPAR-RNAi and empty vector) cell lines. Western blot images and corresponding histogram profiles of PAR-1 and PI3K/Akt signaling pathway proteins in **(C)** HGC-27 (ncRuPAR and empty vector) and **(D)** MGC-803 (ncRuPAR-RNAi and empty vector) cell lines. β-actin was used as the internal loading control for western blotting. Data from three independent experiments were shown for (C) and (D).

Given the known associations between the seven signaling pathways identified from the IPA analysis of the microarray results and the PI3K/Akt signaling pathway, as well as literature reports of the associations between ncRuPAR, PAR-1, and PI3K/Akt signaling 12-14,17,18, the effect of ncRuPAR expression level on PAR-1 and PI3K/Akt signaling pathway was explored in HGC-27 and MGC-803 gastric cancer cell lines *in vitro* by real-time qPCR (Fig. 6A-B) and western blot analysis (Fig. 6C-D and Supplementary material 2). Upregulation of ncRuPAR resulted in decreased PAR-1 mRNA and protein expression while ncRuPAR knockdown led to elevated PAR-1 mRNA and protein expression in human gastric cancer cells (Fig. 6). Overexpression of ncRuPAR resulted in decreased mRNA and protein levels of PI3K, p-AKT and cyclin D1 in HGC-27 cells, while ncRuPAR downregulation led to their elevated mRNA and protein levels in MGC-803 cells (Fig. 6; p < 0.05). However, ncRuPAR expression had no effect on Bcl-2 mRNA and protein levels (Fig. 6).

## Discussion

In this study, stable ncRuPAR knockdown and overexpression human gastric cancer cell lines were established before examining the effect and molecular mechanism of ncRuPAR. It was observed that ncRuPAR upregulation prevented G1/S phase transition, thus inhibiting human gastric cancer cell proliferation and promoting apoptosis *in vitro*. However, ncRuPAR did not affect the migration and invasion of human gastric cancer cells. An Affymetrix GeneChip microarray was used to identify ncRuPAR target genes in the HGC-27 human gastric cancer cell line. The ncRuPAR target genes of ATM, thrombin, and death receptor signaling pathways that were thought to be enriched from the IPA analysis were validated by qPCR, and the differential expression of all genes were confirmed, except for GNG2 in HGC-27 cells that overexpress ncRuPAR. This suggests the possible involvement of these three signaling pathways in ncRuPAR-regulated apoptosis and proliferation of human gastric cancer cells. Additional experiments such as western blotting would be needed to confirm the qPCR results. These three signaling pathways are known to be associated with PI3K/Akt signaling 21-23. Also considering that the known target of ncRuPAR, PAR-1 12, has been reported to activate PI3K/Akt signaling 13, the roles of PAR-1 and PI3K/Akt in ncRuPAR-regulated gastric cancer cell proliferation and apoptosis were investigated. PAR-1, PI3K and p-Akt levels were downregulated in HGC-27-ncRuPAR cells, indicating that ncRuPAR potentially suppressed PAR-1 expression and subsequent PI3K/Akt activation. Given that cyclin D1 and Bcl-2 are known downstream targets of PI3K/Akt and death receptor signaling, their mRNA and protein levels were also examined. The former was downregulated but there was no change in the expression of the latter. The decreased cyclin D1 levels observed were consistent with the results of ncRuPAR inducing G1/S phase cell cycle arrest and decreased cell proliferation. Taken together, these data suggest the involvement of PAR-1, PI3K/Akt signaling and cyclin D1 in ncRuPAR-mediated gastric cancer cell proliferation and apoptosis. Reduction of PAR-1 expression and inhibition of PI3K/Akt activation in gastric cancer may be one of the possible regulatory mechanisms of ncRuPAR. The possible crosstalk between PAR-1 and the PI3K/Akt signaling pathway should be explored in future studies to better understand the regulatory mechanism of ncRuPAR.

IPA network analysis of the microarray results revealed that seven of the top enriched signaling pathways identified (ATM signaling, P2Y purigenic receptor signaling pathway, 14-3-3-mediated signaling, protein kinase A [PKA] signaling, signaling by Rho family GTPases, thrombin signaling and death receptor signaling) were associated with PI3K/Akt signaling. PI3K/Akt activation by the serine/threonine protein kinase ATM 21 and P2Y purigenic receptor signaling pathways was found to promote tumorigenesis 25. The 14-3-3β/γ protein stimulates PI3K/Akt signaling and promotes cancer cell proliferation, migration, invasion, and metastasis 25-27. PI3K/Akt activation by Rac1, a G-protein from the Rho family, can promote prostate cancer cell migration 28. Thrombin was found to activate PI3K/Akt signaling and increase cyclin D1 expression, which promoted cell proliferation in retinal pigment epithelium 22. PKA can stimulate PI3K/Akt signaling and promote granulosa cell proliferation 29. The death receptor signaling pathway regulates tumor cell apoptosis, invasion, and metastasis via PI3K/Akt activation 23. As we only performed a preliminary validation of three out of the seven signaling pathways identified from IPA analysis, the potential involvement of the other four pathways should be verified in follow-up studies.

PAR-1 is a seven-transmembrane G-protein coupled receptor protein that can activate the PI3K/Akt signaling pathway 13,14,16. PAR-1 inhibition was found to inhibit cell death via PI3K/Akt pathway activation 14,15. In addition, both PAR-1 and PI3K/Akt signaling have been implicated in tumorigenesis in triple negative breast cancer^17^and pancreatic cancer 18. Thus, their potential roles in regulating human gastric cancer cell proliferation and apoptosis were examined in this study. Given the previously reported regulation of PAR-1 protein by the lncRNA ncRuPAR during embryonic development 30, it was speculated that they may be similarly associated in gastric cancer. Consistent with a prior study 12, an inverse correlation between ncRuPAR and PAR-1 expression was noted in human gastric cancer by qPCR and western blotting in the current study. Moreover, upregulation of ncRuPAR was also found to inhibit PI3K/Akt signaling, as evidenced by the reduced levels of PI3K and p-Akt from western blotting.

These results revealed that the lncRNA ncRuPAR can inhibit cell proliferation, cause G1/S cell cycle arrest, and induce the apoptosis of gastric cancer cells, but did not affect the migration and invasion of cells. In addition, we found that PAR-1, the PI3K/Akt pathway, and cyclin D1 may be potential downstream targets of ncRuPAR-mediated regulation of human gastric cancer cell proliferation and apoptosis. These findings demonstrate an association between ncRuPAR, PAR-1, the PI3K/Akt pathway, and cyclin D1 in human gastric cancer cell proliferation and apoptosis and propose these molecules as potential targets in gastric cancer diagnosis and therapy.

## Supplementary Material

Supplementary figures.

## Figures and Tables

**Table 1:** Primer sequences for qRT-PCR

GENE	Primer name	Sequence
GAPDH	Forward	5'- AACAGCCTCAAGATCATCAGC-3'
	Reverse	5'- GGATGATGTTCTGGAGAGCC-3'
NcRuPAR	Forward	5'- GGTCTTTTGAAGGAGAAGAGGT-3'
	Reverse	5'- AATGACTATTTTCTAATGCCCGTC-3'
MAPK8	Forward	5'- CCAGGACTGCAGGAACGAGT-3'
	Reverse	5'- CCACGTTTTCCTTGTAGCCC-3'
SMC3	Forward	5'- GGTAGCCCTTGCTCTGATTT-3'
	Reverse	5'- TCCAGAGCCTGGTCAATTTC-3'
SMC2	Forward	5'- CAGGTGGTTATTGGTGGTAGAA-3'
	Reverse	5'- AGGCCAACAGAACAGAAGAG-3'
FANCD2	Forward	5'- CTGAAGGCCATAGAGGAGATTG-3'
	Reverse	5'- GTAGGGAATGTGGAGGAAGATG-3'
MRE11	Forward	5'- GATGAAGTCCGTGAGGCTATG-3'
	Reverse	5'- AGCCATCTGTTCTGCTAAATCT-3'
SMC1A	Forward	5'- GCACTGGATGGAACCCTATT-3'
	Reverse	5'- AGCGCTCCTTCTTCTCTTTC-3'
PLCB4	Forward	5'- GACCTGGCTGTCTTGAGAATAG-3'
	Reverse	5'- ATGTGTCGATATCCGGCTTG-3'
PLCE1	Forward	5'- GACAGCCCATGGAAGGATAAG-3'
	Reverse	5'- CCGGTGATGTACTGCGATATT-3'
PPP1R12A	Forward	5'- TCCCAGACTTTCCTCCTCTT-3'
	Reverse	5'- CTGGCTAGTCGTCTTGGTATTG-3'
PIK3C2A	Forward	5'- AAAGTAAGGCAGGCTAGTGG-3'
	Reverse	5'- GAGAGGGAGACGGCATTTATT-3'
PRKD3	Forward	5'- TCTCTGCCCGACTCTCTAAT-3'
	Reverse	5'- TCCTGGGCTTCAATGGTAAC-3'
GNG12	Forward	5'- CAATATAGCCCAGGCAAGGAG-3'
	Reverse	5'- CTGGCATGTTCCTCACAGTAG-3'
GNG2	Forward	5'- ACAACACCGCCAGCATAG-3'
	Reverse	5'- GCTTCACAGTAGGCCATCAA-3'
TIPARP	Forward	5'- CCACCCTCTAGCAATGTCAA-3'
	Reverse	5'- CGAATGACAGACTCGGGATAC-3'
TNKS2	Forward	5'- CTGAGCCAACCATCCGAAATA-3'
	Reverse	5'- CCACTCCTGGCACTTTCTAAG-3'
MAP4K4	Forward	5'- TGGAGCAGGTTGATGGATATG-3'
	Reverse	5'- TGGAGCATACTTGGCATACG-3'
PAR-1	Forward	5'- GAAACAATCATGGGCGACAAG-3'
	Reverse	5'- CGCTTGGGATCGGCTAAA-3'
PI3K	Forward	5'- GAGTTGGACCTGAGCAATGT-3'
	Reverse	5'- GTCTAACCCGCTCTCGTTTAC-3'
AKT1	Forward	5'- CATTTCTGCTCTGGGCTATCT-3'
	Reverse	5'- CCGAAGTCTGCGACCTTTAT-3'
CCND1	Forward	5'- AAATGCCACTGACTGAGGATAC-3'
	Reverse	5'- GGAGGGTGGATTGGAGATAAAC-3'
BCL-2	Forward	5'- GTGGATGACTGAGTACCTGAAC-3'
	Reverse	5'- GAGACAGCCAGGAGAAATCAA-3'

**Table 2:** The fold-change values from both microarray and qPCR validation

GENE	Microarray fold-change	QPCR fold-change
MAPK8	1.887	1.240 ± 0.064
SMC3	-1.910	-2.197 ± 0.064
SMC2	-2.034	-6.408 ± 2.255
FANCD2	-1.758	-1.512 ± 0.053
MRE11	-1.562	-2.266 ± 0.122
SMC1A	-1.933	-2.222 ± 0.151
PLCB4	-1.655	-1.339 ± 0.434
PLCE1	-1.668	-1.799 ± 0.286
PPP1R12A	-1.784	-2.521 ± 0.692
PIK3C2A	-1.553	-3.138 ± 1.143
PRKD3	-1.529	-5.015 ± 2.306
GNG12	-1.581	-2.423 ± 0.203
GNG2	1.832	-1.274 ± 0.082
TIPARP	1.984	5.174 ± 0.616
TNKS2	3.893	1.831 ± 0.123
MAP4K4	2.268	2.671 ± 1.684
