# Supplementary material for: Long non-coding RNA ncRuPAR regulates gastric cancer cell proliferation and apoptosis via phosphoinositide 3-kinase/protein kinase B signaling
Source: Int J Med Sci. 2022 Oct 17;19(13):1835–46. doi: 10.7150/ijms.76664 (PMC9682512; doi:10.7150/ijms.76664)
Supplement: Supplementary file 1 — Supplementary figures. [file ijmsv19p1835s1.pdf]

Supplementary material 1

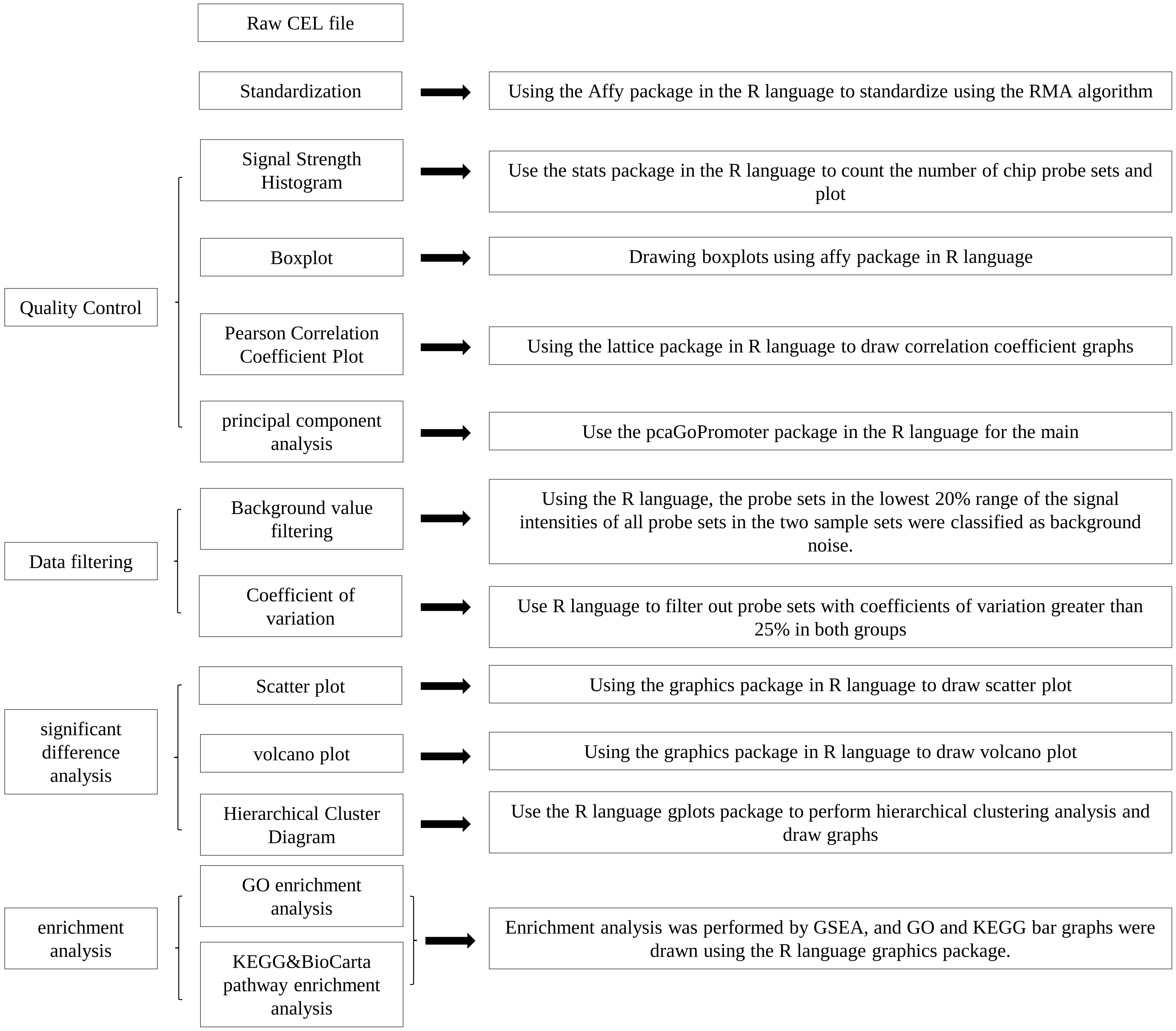

- The expression profile chip was differentially analyzed by the limma program package.
- The 1V1 project cannot calculate the P value, so the volcano map is not provided; the PCA map and the cluster map of the 1V1 project are meaningless, so they are not provided.
- Enrichment analysis: If there is IPA analysis in this section, replace it with IPA analysis.

Supplementary 2

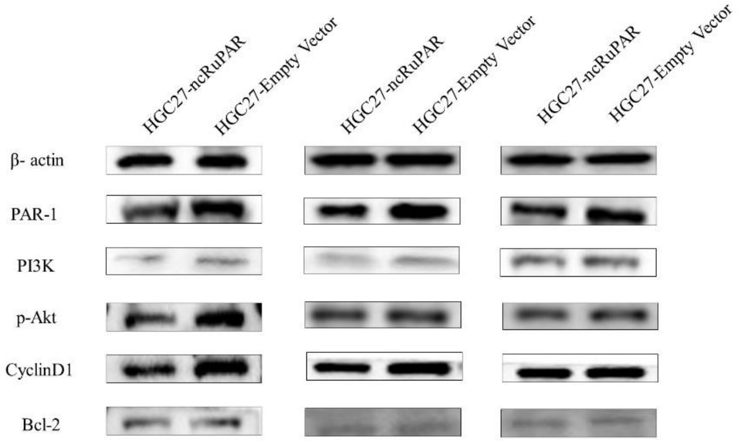

Protein	HGC27-ncRuPAR		HGC27-Empty Vector		P
	Mean	SD	Mean	SD	
PAR-1	0.886	0.149	1.198	0.151	0.018
PI3K	0.308	0.062	0.393	0.067	0.043
p-Akt	0.737	0.109	0.983	0.194	0.021
CyclinD1	0.781	0.140	1.026	0.203	0.023
Bcl-2	0.584	0.095	0.679	0.120	0.319

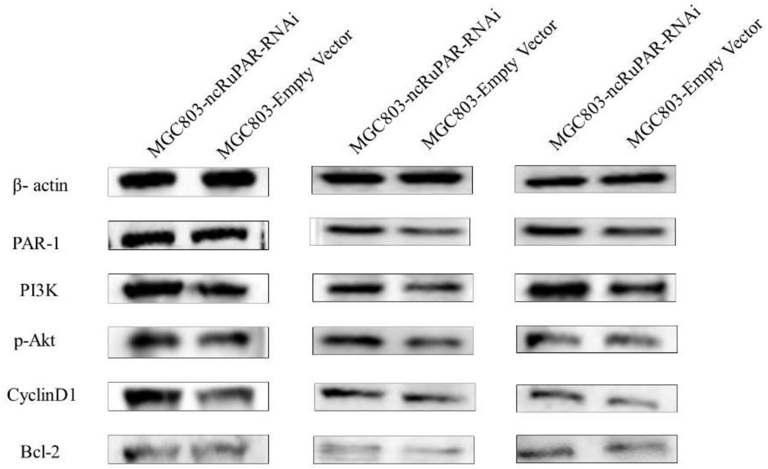

Protein	MGC803-ncRuPAR-RNAi		MGC803-Empty Vector		P
	Mean	SD	Mean	SD	
PAR-1	0.838	0.137	0.640	0.117	0.044
PI3K	1.129	0.286	0.728	0.139	0.031
p-Akt	0.940	0.047	0.639	0.124	0.050
CyclinD1	0.768	0.183	0.494	0.147	0.022
Bcl-2	0.632	0.158	0.531	0.216	0.311
